# Fully automated three‐dimensional deep learning‐based magnetic resonance imaging segmentation of brain cavities in epilepsy surgery

**DOI:** 10.1002/epi.70296

**Published:** 2026-06-12

**Authors:** Raphael Fernandes Casseb, Brunno Machado de Campos, Wallace Souza Loos, Marcelo Eduardo Ramos Barbosa, Marina Koutsodontis Machado Alvim, Gabriel Chagas Lutfala Paulino, Enrico Ghizoni, Francesco Pucci, Samuel Worrell, Clarissa Lin Yassuda, Roberto Medeiros de Souza, Lara Jehi, Fernando Cendes

**Affiliations:** ^1^ Neuroimaging Laboratory Universidade Estadual de Campinas Campinas São Paulo Brazil; ^2^ Advanced Imaging and Artificial Intelligence Lab University of Calgary Calgary Alberta Canada; ^3^ School of Medical Sciences Pontifical Catholic University of Campinas Campinas São Paulo Brazil; ^4^ Department of Neurology Cleveland Clinic Foundation Cleveland Ohio USA

**Keywords:** brain region labeling, nnU‐net, postoperative MRI, ResectVol DL, volumetry

## Abstract

**Objective:**

There are several clinical and research applications for determining the amount of brain tissue resected after epilepsy surgery; however, manual segmentation of postoperative magnetic resonance imaging (MRI) is imprecise and time‐consuming. In this study, we developed and benchmarked ResectVol DL, a freely available deep learning‐based tool that performs this task automatically.

**Methods:**

To create ResectVol DL, we trained a UNet‐like deep learning model using postoperative T1‐weighted MRI from epilepsy surgery patients and evaluated it against manual delineations (ground truth). ResectVol DL was also compared with three automated methods (ResectVol 1.1.2, DeepResection, and Auto3DSeg) using Dice similarity coefficient (DSC), Pearson correlation coefficient, and relative volume difference from manual segmentation. To assess false‐positive detections and generalizability beyond epilepsy, we additionally processed images from healthy controls (no resection) and brain tumor cases.

**Results:**

The final epilepsy cohort comprised 120 patients (57 women, mean age at surgery = 31.5 ± 15.9 [SD] years), split into training (*n* = 72) and test (*n* = 48) sets. An additional 42 images (22 healthy controls and 20 brain tumor cases) were included to test for false positives and generalizability. Segmentation performance differed across methods (Friedman test, *p* < .001). ResectVol Dl achieved the highest median DSC (.925), significantly outperforming ResectVol 1.1.2, DeepResection, and Auto3DSeg after Bonferroni correction. Volume‐based metrics were similar for Auto3DSeg and ResectVol DL (*r* = .988, relative difference = 8.4% vs. *r* = .985, 8.1%; no significant difference), yet Auto3DSeg produced three false‐positive cavities in no‐surgery controls (3/22, 95% confidence interval [CI] = 3%–35%), whereas none was observed for ResectVol DL and DeepResection (0/22, 95% CI = 0%–15%).

**Significance:**

ResectVol DL provides accurate, fully automated segmentation of postoperative resection cavities, offering a robust and reproducible methodological tool for large‐scale postoperative imaging studies in epilepsy surgery. ResectVol DL also provides volumetric information derived from region labeling, which may serve as potential input for predictive models associated with surgery outcome; however, this application has not yet been validated.

**Key points**

- ResectVol DL automates 3D MRI segmentation of brain cavities after epilepsy surgery with high accuracy.
- ResectVol DL achieved a median Dice coefficient of .925, significantly outperforming existing free tools.
- ResectVol DL requires only postoperative MRI, eliminating the need for preoperative scans or manual input.
- ResectVol DL showed no false positives in healthy control images without resections.
- Strong generalizability to non‐epilepsy resective cases was shown (DSC = .907 in postoperative images of brain tumor patients).

## 1 INTRODUCTION

Epilepsy has a profound impact on quality of life, morbidity, and mortality. Approximately 30%–40% of patients do not respond to treatment with medication^1^, ^2^ and can be referred to surgical intervention, especially if structural alterations are detected.^3^, ^4^ Nevertheless, up to 50% of those who undergo brain surgery do not achieve sustained seizure freedom.^5^ Therefore, it is crucial to identify factors that can enhance planning to improve the efficacy of surgical treatment.

Studies have tried to determine the factors leading to surgical success,^6^, ^7^, ^8^ and nomograms were created showing promising results for individualizing outcome prediction.^9^, ^10^ However, most surgical outcome studies still categorize the surgical procedure under broad classifications, with resolution limited to the lobe of resection (temporal/extratemporal, hippocampal sparing vs. hippocampal resection). This oversimplification may dismiss important knowledge that could be imparted to predictive models, such as the size or volume of parts removed (e.g., 90% of the hippocampus, 30% of the superior temporal gyrus) instead of simply labeling them as “temporal” or “extratemporal.”

Manual delineation of the cavity can be used to characterize resections by providing volumetric information and supporting further investigations. Although this assessment is not typically time‐critical in a clinical setting, it is labor‐intensive, increasing experts' workload and limiting scalability in larger projects or studies. In addition, when a large number of segmentations is needed in a research context, the time required per subject segmentation is very relevant. Therefore, efforts to automate this task have increased in recent years, leading to the development of deep learning and rule‐based tools.^11^, ^12^, ^13^, ^14^, ^15^ ResectVol 1.1.2^12^ and EPIC‐Chop^14^ are methods based on the Statistical Parametric Mapping toolbox^16^ that use a sequence of processing steps to automatically obtain the cavity delineation. Considering that the cavity appears as a dark region on T1‐weighted magnetic resonance imaging (MRI), with absent gray and white matter, the rationale behind both tools ultimately relies on subtracting the postoperative image from the preoperative one. In general, rule‐based methods tend to be slow, given the time required to run each of the several pipeline steps, and parameters set to process data may work for a group of images, but not all of them (e.g., parameters set to perform brain extraction may not work for all images in the dataset). Methods based on deep learning, such as Resseg^15^ and DeepResection,^13^ rely on the training of neural networks to segment the dark, cerebrospinal fluid‐filled regions in brain MRIs that are not ventricles or other natural cavities. They tend to be more accurate than rule‐based alternatives but often require large and diverse datasets. Data augmentation strategies and foundational models have helped address this issue, but they have limitations. The authors of the DeepResection tool, for instance, noted that training the network with the aid of data augmentation on temporal resective data and fine‐tuning it with extratemporal cases was insufficient to achieve good performance on the extratemporal test set.^13^ In line with this, despite the prestige deep learning has gained, a recent comparison of cavity segmentation methods^17^ showed that rule‐based methods were able to detect more cavities (44 of 50) than their deep learning counterparts (23 of 50). Nevertheless, to the best of our knowledge, the current deep learning tools do not use the nnU‐Net framework. This three‐dimensional (3D) self‐adapting architecture was recently featured among the best segmentation methods when compared to more modern architectures, such as Transformer‐based and Mamba‐based models.^18^

In this context, the purpose of this study is twofold: (1) to introduce ResectVol DL, a fully automatic deep learning method for segmenting temporal and extratemporal brain cavities based on nnU‐Net^19^; and (2) to compare ResectVol DL against three other tools: ResectVol 1.1.2, DeepResection, and Auto3DSeg.^20^ ResectVol 1.1.2 and DeepResection were identified as the most sensitive rule‐based and deep learning‐based methods, respectively, for cavity segmentation.^17^ Auto3DSeg, part of the MONAI ecosystem of artificial intelligence tools,^21^ is a self‐configuring framework that integrates multiple state‐of‐the‐art deep learning architectures and has achieved top rankings in medical image segmentation challenges.

## 2 MATERIALS AND METHODS

This study was conducted with approval from the Cleveland Clinical Foundation Institutional Review Board. Informed consent was waived due to the retrospective nature of the deidentified MRI data collection.

### 2.1 Subjects and image datasets

Image datasets from patients with epilepsy were retrospectively selected from the Cleveland Clinic Epilepsy Center and the University of Campinas Neuroimaging Laboratory, comprising individuals with temporal and extratemporal lobe epilepsy. Additionally, 22 healthy volunteers were included to serve as controls for false discoveries (no‐surgery cases), and patients with brain tumors who were treated with resective surgery were added to evaluate the generalizability of our tool to non‐epilepsy cases. Only cases involving resective surgeries were included.

### 2.2 Manual segmentation

All postoperative images containing brain cavities were manually segmented in MRIcron^22^ to provide ground truth segmentation masks. Images that were segmented more than once were used to estimate interrater agreement.

### 2.3 Automatic segmentation

**FIGURE 1:**
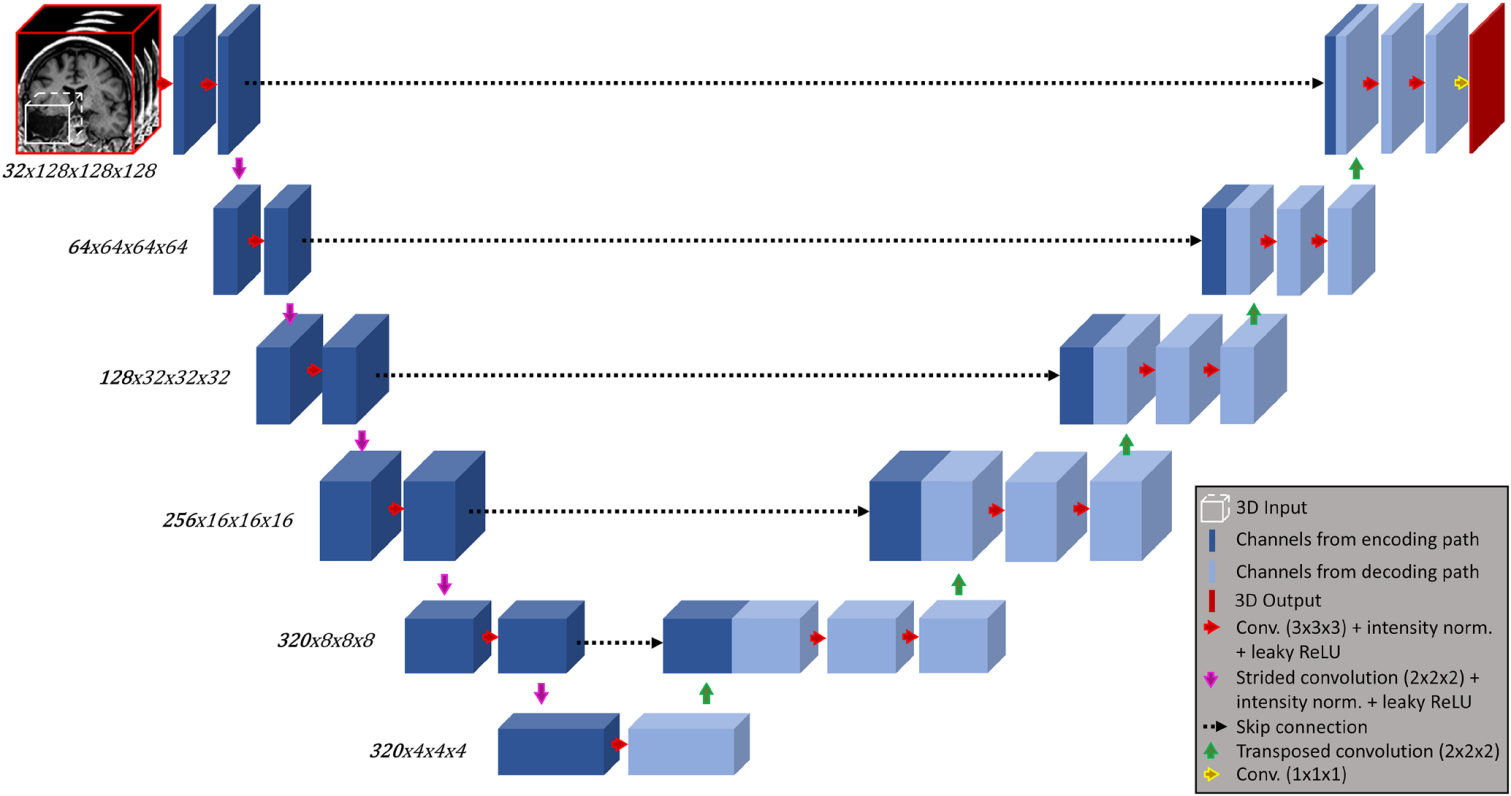
Representation of the three‐dimensional (3D) nnU‐Net model. The number of channels and resolution at each of the six stages are shown on the left (e.g., there are 320 channels and a resolution of 4 × 4 × 4 in the feature map of the sixth stage). Contiguous blocks after the green arrows represent the concatenation of channels from the lower stage of the decoding branch (light blue) with channels at the same stage from the encoding branch (dark blue). Conv., convolution; norm., normalization; ReLu, rectifying linear unit.

To perform automatic segmentation, we developed ResectVol DL, a program based on the nnU‐Net deep learning framework,^23^ itself derived from the U‐Net architecture.^19^ This widely used neural network consists of an encoder, a decoder, and skip connections that transfer features from the encoder to the decoder. In general, each stage in the encoding and decoding streams of a U‐Net‐like architecture combines convolution steps, intensity normalization, and a nonlinear function (e.g., the rectifying linear unit or ReLU). The specific processing steps are displayed in Figure 1, along with other parameters that are automatically defined by the framework.

To increase data variability, augmentation strategies are applied using the *batchgenerators* Python package. These augmentations included rotation (*p* = .16, *U*[−30°, −30°]), scaling (*p* = .16, zoom ∼ *U*[.7, 1.4]), rotation + scaling (*p* = .08), addition of Gaussian noise (*p* = .15, voxelwise, variance ∼ *U*[0, .1]), Gaussian blurring (*p* = .2, kernel width ∼ *U*[.5, 1.5]), changes to brightness (*p* = .15, multiplication factor ∼ *U*[.7, 1.3]) and contrast (*p* = .15, multiplication factor ∼ *U*[.65, 1.5]), gamma augmentation (*p* = .15, gamma factor ∼ *U*[.7, 1.5]), low‐resolution simulation (*p* = .25, downsampling factor ∼ *U*[1, 2]), and mirroring (*p* = .5, along all axes).^24^ The notation *c* ∼ *U*(*a*, *b*) means that the factor *c* is drawn from a uniform distribution with minimum value *a* and maximum *b*.

Model training was conducted with five cross‐validation folds and 1000 epochs per fold, utilizing the sum of Dice and cross‐entropy as the loss function to optimize both the overlap between predicted and ground‐truth segmentations and voxelwise classification accuracy. The dataset was split at the patient level to prevent data leakage across the training, validation, and test sets. Training was performed using stochastic gradient descent with Nesterov momentum (*μ* = .99), starting with an initial learning rate of .01 that decayed over time following a polynomial schedule. During inference, nnU‐Net averages the softmax probability outputs from the models trained on each fold, creating an ensemble prediction. The final segmentation masks are obtained by applying an argmax operation over the averaged softmax probabilities. In the context of our binary segmentation task, this approach is effectively equivalent to thresholding at a probability of .5.^23^

The nnU‐Net framework simplifies the training process of the 3D U‐Net by automatically adjusting architectural and training parameters based on data characteristics. ResectVol DL incorporates the resulting architecture and weights as its core algorithm, integrating functionalities from other tools to label and estimate the volume of surgically removed brain structures (see Appendix S1).^25^, ^26^, ^27^

Users can execute segmentation and labeling via simple command‐line instructions. ResectVol DL saves masks of the entire cavity and each identified structure in separate compressed files in the NIfTI (Neuroimaging Informatics Technology Initiative) format (.nii), which can be used, for instance, as regions of interest for functional connectivity studies. Additionally, it generates a text file with the estimated volume of the resected brain structures.

To compare our algorithm against other freely available methods, we also performed the cavity prediction with three other tools: ResectVol version 1.1.2 (available at lniunicamp.com/resectvol), a rule‐based algorithm previously developed by our group that does not employ deep learning models^12^; the DeepResection tool (available at github.com/penn‐cnt/DeepResection),^13^ which uses a set of three 2D U‐Nets, each in one anatomical plane, followed by voxelwise majority voting; and Auto3DSeg (version 1.4.0),^20^, ^21^ a framework that allows for the training of three different models—DiNTS,^28^ SegResNet,^29^ and Swin UNETR^30^—to create a segmentation ensemble of the best performing ones. In ResectVol 1.1.2, users must provide both pre‐ and postoperative images to run the analysis, whereas DeepResection and Auto3DSeg require only the postoperative image.

### 2.4 Computational resources

All automatic processing was performed on a 3.6‐GHz Intel Core i7‐12700KF CPU with 32 GB RAM, a 48 GB NVIDIA RTX A6000, and a 20.04.1 Ubuntu operating system.

### 2.5 Performance assessment and statistical analysis

The Dice similarity coefficient (DSC)^31^ was used as an indirect measure of overlap between manual and automatic segmentations. Segmentation failure was defined based on the conservative criterion of DSC = 0 (no overlap between the automated and manual masks). We also calculated the volumes obtained from the manual and automatic segmentations, and estimated Pearson correlation coefficient (*r*) and the percentage difference in volume (manual volume as the reference) between them.

Statistical analyses were performed using a repeated‐measures framework, as all segmentation methods were applied to the same subjects. DSC and relative volumetric differences were calculated on a per‐subject basis and summarized as medians with interquartile ranges (first quartile [Q1]–third quartile [Q3]). Overall differences across methods were assessed using the Friedman test. When significant, pairwise comparisons were performed using Wilcoxon signed‐rank tests with Bonferroni correction for multiple comparisons. Effect sizes were reported as median paired differences with 95% confidence intervals (CIs) obtained by bootstrap resampling of subjects (20 000 iterations).

Associations between automatically estimated and manually segmented resection volumes were evaluated using Pearson correlation coefficient, with 95% CIs derived using Fisher *z* transformation. We use nonparametric bootstrap resampling of subjects (20 000 iterations) to estimate CIs and *p*‐values for the difference in correlations (Δ*r*), with CIs and two‐sided *p*‐values obtained by evaluating whether the bootstrap distribution of Δ*r* included zero. Additionally, to investigate whether segmentation performance was associated with cavity size, Spearman rank correlation was computed between DSC and cavity volume. For false‐positive detections on the no‐surgery, healthy control cases, we calculated false‐positive rates with 95% Clopper–Pearson CIs, and pairwise differences between methods were assessed using McNemar exact test with Bonferroni correction for multiple comparisons.

Regarding the interrater segmentation performance, we calculated the DSC for the subset of extratemporal images that were manually segmented more than once (Appendix S3), because extratemporal resections are more variable in location, complexity, and size than temporal ones.

## 3 RESULTS

### 3.1 Subjects

The epilepsy cohort comprised 120 patients (57 women, mean age = 31.5 ± 15.9 [SD] years at surgery), including 45 with temporal (23 women, mean age = 37.7 ± 13.8 [SD] years) and 75 with extratemporal (34 women, mean age = 27.8 ± 16.0 [SD] years) epilepsy. Nearly all postoperative images were acquired ≥5 months after surgery (median = 6.2, Q1–Q3 = 6.0–8.0, range = .2–63.9), to avoid necrotic tissue that could be misleading in the segmentation process. Seventy‐three patients (~61%) were seizure‐free (Engel IA) after surgery.

The no‐surgery control cohort comprised 22 healthy subjects (14 women, mean age = 28.8 ± 4.0 [SD] years). Twenty individuals (eight women, mean age = 36.0 ± 13.1 [SD] years) who underwent surgical removal of brain tumor were also included to test the generalizability of the tool in a different disease context. Images for both non‐epilepsy cohorts (healthy controls and brain tumor cases) were retrieved from the Campinas center.

Each surgical participant contributed one postoperative magnetic resonance (MR) image, resulting in a total of 162 postoperative MRIs. For the set of 70 participants used in the comparative tool testing—comprising 48 epilepsy cases and 22 healthy controls—corresponding preoperative images were also included (for healthy controls, “preoperative image” refers simply to an image acquired at an earlier time point), as one of the reference methods (ResectVol 1.1.2) required both pre‐ and postoperative scans for analysis (Table 1).

**TABLE 1:** Summary of the number of images and their specific use (training, testing, specificity, or generalizability assessment) for each group of subjects.

Group	Number of participants	Dataset division	Description
Temporal epilepsy	45	Training: 22 postop Test: 23 postop + 23 preop^a^	Patients with resective surgery for temporal epilepsy
Extratemporal epilepsy	75	Training: 50 postop Test: 25 postop + 25 preop^a^	Patients with resective surgery for extratemporal epilepsy
Healthy controls	22	Specificity test: 22 postop + 22 preop^a^	No brain lesions or resective interventions; tested for false positives
Brain tumor	20	Generalizability test (ResectVol DL only): 20 postop	Resective surgeries unrelated to epilepsy; tested for generalizability
Total	162	Training: 72 postop Test: 90 postop + 70 preop^a^	Used in training or testing

Abbreviations: postop, postoperative image; preop, preoperative image.

^a^ ResectVol 1.1.2 requires both preop and postop images to perform the segmentation.

### 3.2 Imaging protocol

T1‐weighted anatomical MR images were obtained in a total of eight different MRI systems from two distinct vendors (Siemens and Philips). Forty‐four subjects were scanned in a 1.5‐T MR device, whereas 118 individuals were scanned in a 3‐T MR machine. Acquisition parameters and image quality varied, benefiting the robustness of the performance assessment. Parameters can be summarized with the following ranges: reconstructed voxel size = .41 × .45 × .41 to 1 × 2 × 1 mm^3^, repetition time = 7–2200 ms, echo time = 2.3–5.0 ms, and image matrix = 180 × 96 to 512 × 512. Table S2 lists the acquisition protocol for the 162 scanned subjects.

### 3.3 ResectVol DL

Seventy‐two images were used to train the nnU‐Net model (*n*_Campinas_ = 21, *n*_Cleveland_ = 51). We found a median DSC of .905 (Q1–Q3 = .848–.932) and .925 (Q1–Q3 = .882–.945) for the cross‐validation and test sets, respectively (Table 2).

**TABLE 2:** DSC, correlation, and relative difference obtained with each method for the test sets.

Test set	ResectVol DL	ResectVol 1.1.2	DeepResection	Auto3DSeg
All epilepsy data, *n* = 48				
Median DSC, Q1–Q3	.**925 (.882–.945)** ^a^	.775 (.651–.835)^a^	.072 (0–.895)^b^	.919 (.883–.945)^a^
Range	0–.964	0–.899	0–.947^b^	0–.967
Pearson *r* [95% CI]^c^	.985 [.973–.992]^d^	.628 [.419–.774]^d^, ^e^	‐	.988 [.979–.994]^e^
Median rel. diff. (Q1–Q3)	8.1 (3.7–12.7)%^d^	44.0 (29.4–74.6)%^d^, ^e^	‐	8.4 (3.3–16.8)%^e^
Temporal only, *n* = 23				
Median DSC (Q1–Q3)	.**937 (.895–.951)** ^a^	.794 (.752–.855)^a^	.898 (.858–.919)^a^	.932 (.894–.947)^a^
Range	.850–.963	.213–.899	.198–.947	.299–.955
Pearson *r* [95% CI]^c^	.979 [.951–.991]^d^	.717 [.433–.872]^d^, ^e^	.940 [.862–.975]	.968 [.925–.987]^e^
Median rel. diff. (Q1–Q3)	5.5 (3.6–9.4)%^d^, ^f^	44.9 (29.7–65.1)%^d^, ^e^, ^g^	12.5 (6.3–19.6)%^f^, ^g^, ^h^	8.5 (3.8–15.2)%^e^, ^h^
Extratemporal only, *n* = 25				
Median DSC (Q1–Q3)	.**919 (.865–.936)** ^d^	.729 (.607–.790)^d^, ^e^	0 (0–0)^b^	.**919 (.870–.941)** ^e^
Range	0–.964	0–.891	0–.08^b^	0–.967
Pearson *r* [95% CI]^c^	.990 [.976–.995]	.619 [.296–.815]^e^	‐	.995 [.988–.998]^e^
Median rel. diff. (Q1–Q3)	11.2 (4.5–17.4)%^d^	36.7 (14.8–83.1)%^d^, ^e^	‐	8.4 (2.6–16.7)%^e^
Brain tumor, *n* = 20				
Median DSC (Q1–Q3)	.907 (.840–.952)	‐	‐	‐
Range	.673–.965	‐	‐	‐
Pearson *r* [95% CI]^c^	.992 [.980–.997]	‐	‐	‐
Median rel. diff. (Q1–Q3)	10.4 (4.5–18.3)%	‐	‐	‐
Healthy controls, *n* = 22				
False‐positive rate, *n*/22 (%) [95% CI]^j^	**0/22 (0) [.00–15.44]** ^d^	22/22 (100) [84.56–100.00]^d^, ^e^, ^f^	**0/22 (0) [.00–15.44]** ^g^	3/22 (13.64) [2.91–34.91]^e^

*Note*: DeepResection results are not considered for the analyses of “all epilepsy” and “extratemporal only” data, as recommended by DeepResection authors. Bold values indicate the highest median DSC for each disease group and the lowest false‐positive rate among healthy controls.

Abbreviations: CI, confidence interval; DSC, Dice similarity coefficient; Q1, first quartile; Q3, third quartile; rel. diff., relative difference.

^a^ Significant pairwise comparisons (Bonferroni‐corrected) across all applicable pairs.

^b^ The authors do not recommend applying DeepResection to extratemporal cases. “All epilepsy” and “extratemporal only” metrics values are only reported for completeness. The associated correlation values, however, are not displayed due to the impossibility related to data distribution (see Figure S2A, blue dots in the DeepResection plot).

^c^ CI for Pearson correlation coefficient (*r*) was derived using Fisher *z* transform.

^d^ Significant pairwise comparison (Bonferroni‐corrected): ResectVol DL–ResectVol 1.1.2.

^e^ Significant pairwise comparison (Bonferroni‐corrected): Auto3DSeg–ResectVol 1.1.2.

^f^ Significant pairwise comparison (Bonferroni‐corrected): ResectVol DL–DeepResection.

^g^ Significant pairwise comparison (Bonferroni‐corrected): ResectVol 1.1.2–DeepResection.

^h^ Significant pairwise comparison (Bonferroni‐corrected): Auto3DSeg–DeepResection.

^i^ Significant pairwise comparison (Bonferroni‐corrected): ResectVol DL–Auto3DSeg.

^j^ Clopper–Pearson exact CI.

Segmentation performance differed significantly across methods (Friedman test for repeated measures, *p* < .001) when comparing paired differences. Post hoc Wilcoxon tests (Bonferroni‐corrected for multiple comparisons) showed that ResectVol DL achieved a higher DSC than ResectVol 1.1.2 in the three groups: all epilepsy cases (median ΔDSC = .127, *p*_Bonf_ < .001) and in both temporal‐only (median ΔDSC = .122, *p*_Bonf_ < .001) and extratemporal‐only analyses (median ΔDSC = .135, *p*_Bonf_ < .001; Table S3). ResectVol DL also outperformed DeepResection in temporal cases (median ΔDSC = .029, *p*_Bonf_ < .001), whereas results for the other datasets (all and extratemporal cases) could not be estimated due to DeepResection's limitation on extratemporal data. Compared with Auto3DSeg, ResectVol DL showed a small but statistically significant DSC difference in all epilepsy cases (median ΔDSC = .004, *p*_Bonf_ = .024) and in temporal‐only cases (median ΔDSC = .005, *p*_Bonf_ = .022), whereas performance was comparable in extratemporal cases (median ΔDSC = .003, *p*_Bonf_ = 1).

For 47 of the 48 test images (*n*_Campinas_ = 9, *n*_Cleveland_ = 39), ResectVol DL was able to correctly identify the cavity and failed for only one extratemporal case—the smallest cavity in the epilepsy test set (494.6 mm^3^)—for which no cavity was detected. The correlation between the manual and the automatic volumes was *r*(46) = .985 (*p* < .001), with a median relative difference of 8.1% (Q1–Q3 = 3.7%–12.7%; Table 2 and Figure S2). Correlation strength did not differ from Auto3DSeg (Δ*r* = −.004, *p*_Bonf_ = 1), whereas ResectVol DL showed statistically higher correlation than ResectVol 1.1.2 (Δ*r* = .357, *p*_Bonf_ = .0024; Table S5). Regarding relative volumetric differences, ResectVol DL exhibited no difference from Auto3DSeg (ΔRelDiff = .0, *p*_Bonf_ = 1), but outperformed ResectVol 1.1.2 (ΔRelDiff = − 32.1, *p*_Bonf_ < .001; Table S4).

ResectVol DL (and DeepResection) correctly identified the absence of cavities in all 22 healthy control datasets (0/22, 0% [95% CI = 0%–15%]), whereas Auto3DSeg produced false‐positive cavities in three of 22 controls (14% [95% CI = 3%–35%]). This difference was not statistically significant (McNemar exact *p* = .25). In contrast, ResectVol 1.1.2 produced false positives in all 22 of 22 controls (100% [95% CI = 85%–100%]), significantly worse than ResectVol DL (McNemar exact *p*_Bonf_ < .001). Regarding the brain tumor images, ResectVol DL achieved a median DSC of .907 (95% CI = .840–.952). The prediction for each image took, on average, 40.8 s.

**FIGURE 2:**
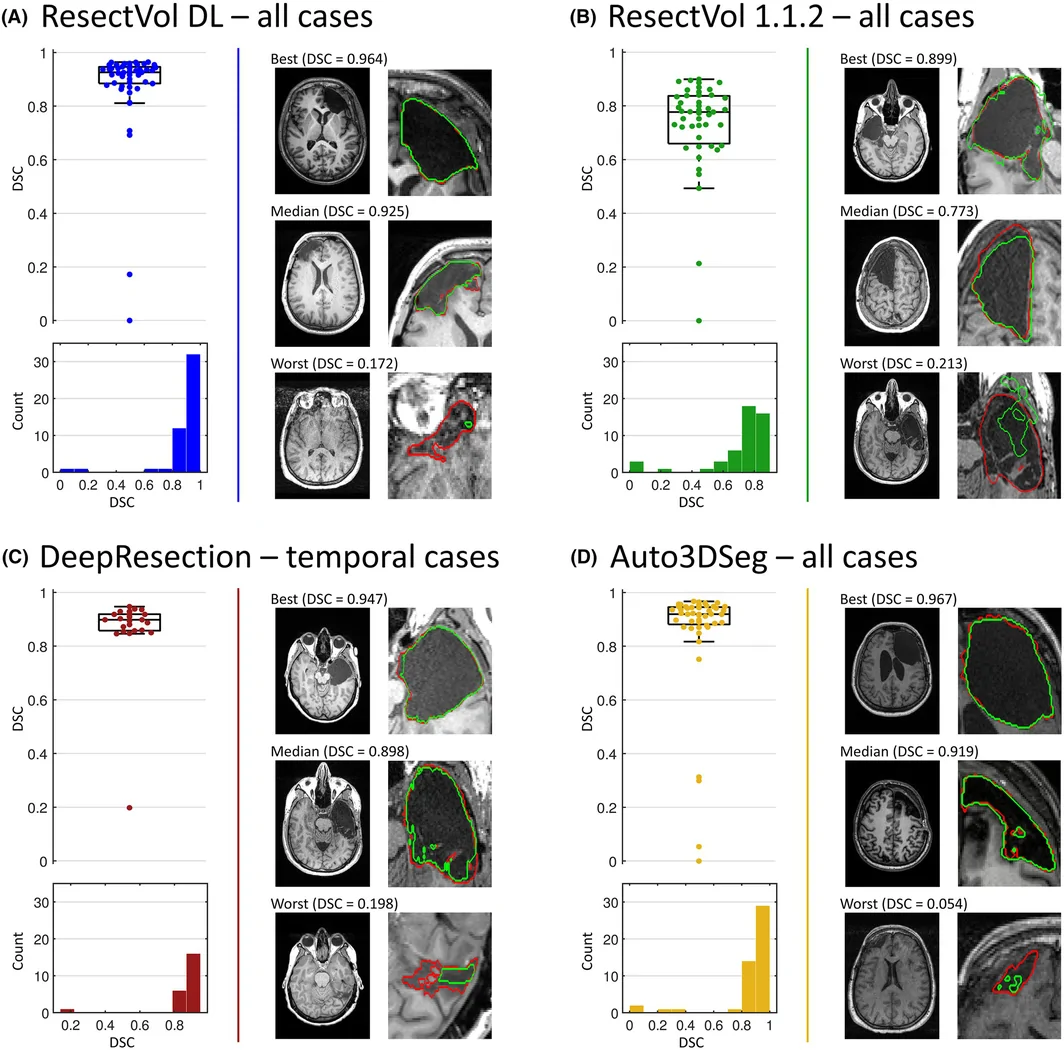
Boxplots, histograms, and noteworthy cases for (A) ResectVol DL, (B) ResectVol 1.1.2, (C) DeepResection, and (D) Auto3DSeg, along with Dice similarity coefficient (DSC). Red and green contours represent manual and automatic segmentation. ResectVol DL (A), ResectVol 1.1.2 (B), and Auto3DSeg (D) were applied to temporal and extratemporal data (*n* = 48), whereas DeepResection (C) was only applied to temporal cases (*n* = 23) due to its limitation on extratemporal data. Although included in boxplots and histograms, cases with DSC = 0 are not shown in the figures.

The performance of the other tools is detailed in Appendix S4 and summarized in Table 2. Figure 2 exhibits the best, median, and worst DSC‐associated cases for all tools, and Figure S2 presents the corresponding correlation plots. Tables S3–S5 show the median effect sizes and CIs along with the results of all significance tests of the pairwise comparisons.

**FIGURE 3:**
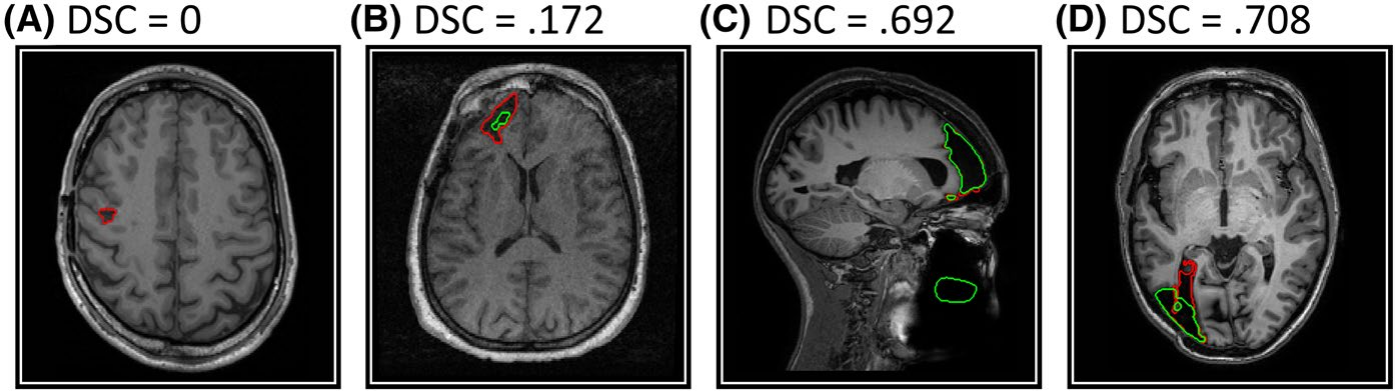
Poor segmentation performance cases for ResectVol DL in the epilepsy test set, assessed with the Dice similarity coefficient (DSC). (A) Smallest cavity in the test set not detected (DSC = 0). (B) Severe noise/low contrast leading to an incomplete delineation (DSC = .172). (C) Mouth region artifact erroneously segmented as a cavity (DSC = .692). (D) Disagreement driven by ventricular inclusion in the manual label versus ventricular sparing by ResectVol DL (DSC = .708).

Four cases yielded DSCs < .80, which we considered poor segmentation performance. For the smallest cavity of the epilepsy test set (494.6 mm^3^), ResectVol DL did not detect a cavity (DSC = 0; Figure 3A), as mentioned previously. In another case, markedly low image quality/noise reduced contrast between the cavity and surrounding tissue and disrupted the typical hypointense appearance of the cavity, leading ResectVol DL to produce a very conservative and incomplete delineation (DSC = .172; Figure 3B). In a third case, an artifact affecting the mouth region, consistent with susceptibility from metal hardware like implants and orthodontic braces, created a hypointense area that was incorrectly identified as a cavity outside of the skull (DSC = .172; Figure 3C). Finally, we observed a delineation divergence due to manual segmentation including the ventricular space, whereas ResectVol DL tended to exclude the ventricle from the resection mask (DSC = .708; Figure 3D).

### 3.4 Large versus small cavities

**FIGURE 4:**
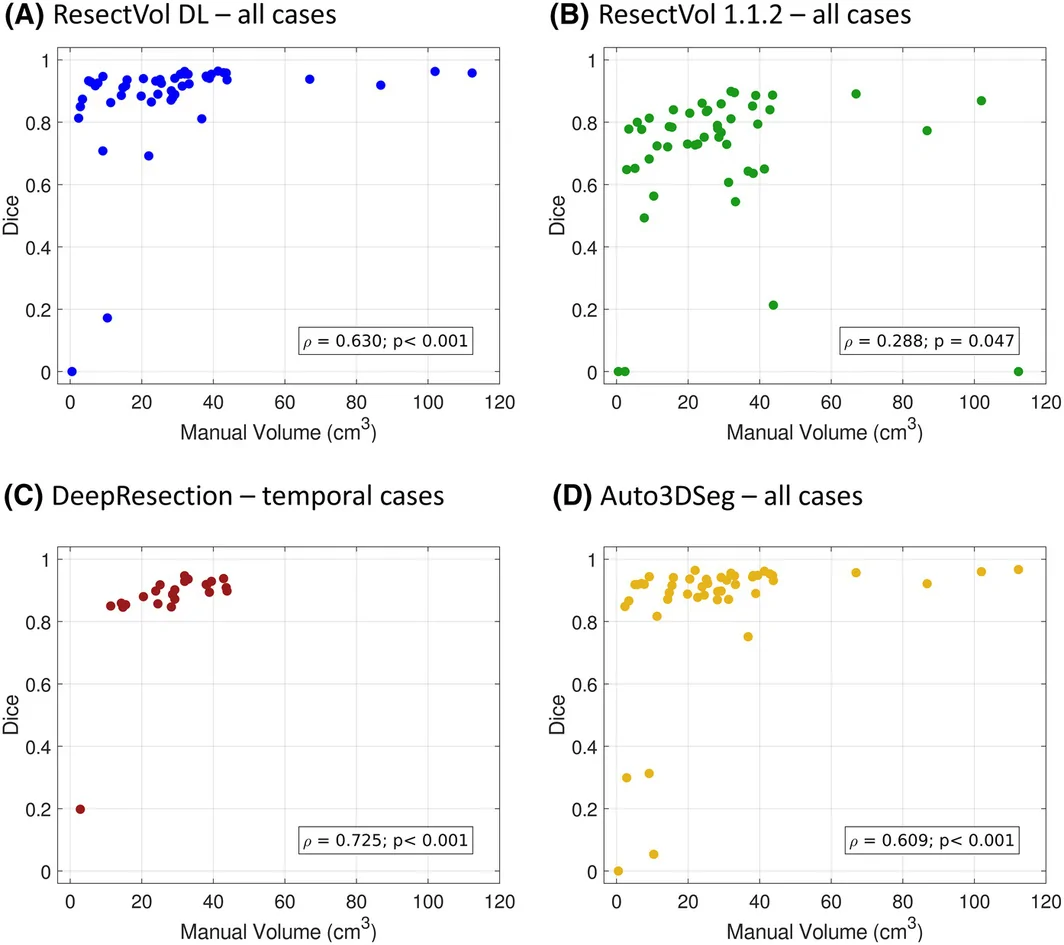
Scatterplots of Dice similarity coefficient versus manual volume (cm^3^) for (A) ResectVol DL, (B) ResectVol 1.1.2, (C) DeepResection, and (D) Auto3DSeg. Text boxes in the bottom right of each plot exhibit the Spearman rank correlation coefficient (*ρ*) and the associated *p*‐value (*p*).

Cavities were segmented both automatically and manually. Results of the interrater agreement analysis are provided in Appendix S3. The Spearman correlation between volume (in cm^3^) and DSCs was statistically different from zero (*p* < .05) for all tools: *ρ*_ResectVol DL_ (46) = .630, *ρ*_ResectVol 1.1.2_ (46) = .288, *ρ*_DeepResection_ (21) = .725, and *ρ*_Auto3DSeg_ (46) = .609. Figure 4 depicts the relationship between cavity volume and the DSC.

## 4 DISCUSSION

In this study, we benchmarked four automatic segmentation methods against the manual delineation of brain cavities on postoperative T1‐weighted MR images. We found that ResectVol DL, built on the nnU‐Net framework, outperformed its previous rule‐based implementation (ResectVol 1.1.2) and the 2D U‐Net‐based DeepResection tool, while performing on par with the self‐configuring Auto3DSeg, which was also trained by us. Courtney et al.^17^ previously identified ResectVol 1.1.2 and DeepResection as the most sensitive rule‐based and deep learning‐based tools for cavity detection. Our findings demonstrate that utilizing an nnU‐Net backbone enhances accuracy, underscoring its potential to improve postoperative assessments in epilepsy surgery. Although Auto3DSeg has excelled in public segmentation challenges and delivered very competitive scores in our study, ResectVol DL matched its accuracy and showed an advantage in producing no false positives. In addition, ResectVol DL includes the volumes of substructures inside the cavity, which can be useful for correlating with postoperative seizure and cognitive outcomes.

When comparing median DSCs for temporal cases among the fully automatic tools we tested, ResectVol DL (DSC = .937) was statistically better than DeepResection (DSC = .898, *p* < .001) and ResectVol 1.1.2 (DSC = .794, *p* < .001), and slightly exceeded Auto3DSeg (DSC = .932, *p* = .022). Although DeepResection is capable of being fine‐tuned to work with extratemporal resections—as tested in the original publication (exploratory DSC = .75)—the authors do not recommend using this preliminary fine‐tuned model. Hence, for extratemporal cases, we only considered the results from the other three tools, and both ResectVol DL (DSC = .919, *p* < .001) and Auto3DSeg (.919, *p* = .006) outperformed ResectVol 1.1.2 (.729). Volume‐based metrics were similar for Auto3DSeg and ResectVol DL (*r* = .988, relative difference = 8.4% vs. *r* = .985, 8.1%; no significant difference), yet Auto3DSeg produced three false‐positive cavities in controls (3/22, 95% CI = 2.9%–34.9%), whereas no false positives were observed for ResectVol DL and DeepResection (0/22, 95% CI = 0%–15.4%). Furthermore, ResectVol DL demonstrated strong performance on brain tumor images (DSC = .907), indicating its potential applicability to other disease contexts beyond epilepsy surgery. Regarding prediction runtime, DeepResection was the fastest (≈35 s per image), followed by Auto3DSeg (≈39 s) and ResectVol DL (≈41 s). ResectVol 1.1.2 took much longer (≈9 min) due to the nature of its processing algorithm, which is based on a series of processing steps.

Deep learning methods excel in complex tasks, such as volumetric segmentation, because they can model intricate, high‐level dependencies within the data. Specifically, ResectVol DL and Auto3DSeg benefit from using a 3D U‐Net‐like architecture,^19^, ^32^ which processes 3D volumetric data, enabling it to learn spatial dependencies across all three dimensions simultaneously. This contrasts with DeepResection's 2D U‐Net approach, which applies segmentation independently in each dimension (anteroposterior, laterolateral, craniocaudal) and then integrates results through majority voting.^13^ This method may miss finer interdimensional relationships, which likely contributes to its lower performance compared to the other 3D‐based tools. Additionally, the use of adapting frameworks further enhances ResectVol DL and Auto3DSeg's capabilities by automating critical aspects of model configuration, such as preprocessing, architecture customization, and hyperparameter tuning. The optimization processes ensure that the model is tailored specifically to the dataset's characteristics, reducing the need for manual intervention and fine‐tuning.^18^

As previously reported in other studies,^13^, ^33^ we found a relationship between DSC and cavity size, indicating that there is a trend toward better performance for larger resections (Figure 4). Because DSC is dependent upon a volume ratio, large values in the numerator (intersection) and denominator (total volume) will be less sensitive to intersection mismatches when comparing two volumetric shapes. Hence, small cavities are more prone to being affected by discrepancies and present more variability in DSCs. Interestingly, the only case for which none of the methods was able to identify any cavity is the dataset with the smallest cavity from the epilepsy test set.

Besides this small cavity with DSC = 0, the other three datasets yielded poor results in ResectVol DL's analyses (DSC < .8), mainly associated with image artifacts and differences in labeling conventions (Figure 3). Not surprisingly, one image with markedly high noise and low quality exhibited a low contrast cavity (Figure 3B) distinct from the hypointense pattern typically expected, leading to an incomplete segmentation. Ideally, such images should be minimized through acquisition quality control, but it was retained in our study to reflect real‐world variability and to identify limitations of our tool. The other artifactual image (Figure 3C), with no signal in the mouth region, generated a segmentation cluster outside of the brain. In practice, this would demand a user intervention by either removing the wrong cluster or by cropping the problematic non‐brain region prior to processing. Including cropping steps at this point in our tool would add complexity and processing overhead for a very specific issue and was therefore not implemented at this time. However, this is another point of caution for users. The third case (Figure 3D) regards a ventricle sparing in the segmentation that is harder to perform manually given the lack of a clear boundary. This is not necessarily an error, but it underscores the need to standardize ventricular inclusion/exclusion criteria, particularly in studies relying on cavity volume as an outcome, to prevent avoidable variability across raters and methods.

In this context of cavity misidentification, semiautomated methods can be a valuable solution, because users must click on a point within the cavity to perform the segmentation.^11^, ^33^ The major advantage of these approaches is that the seed is always positioned in the right location, where the cavity is expected to be, making them extremely efficient in challenging individual cases. Nevertheless, this approach still requires human intervention and is prone to bias (because manual positioning of the seed may yield different results), and, although we did not make a direct comparison on the same dataset, the DSC found for ResectVol DL (.93) is better than in these studies.

In addition to the volumetric information obtained through such computational segmentation methods, this line of research provides a quantitative framework that may, in the future, contribute to improved surgical planning and outcome prediction. For example, Koepp et al.^34^ reported that the removal of at least 50% of the piriform cortex increased (by a factor of 16) the odds of becoming seizure‐free, and no relationship was found with other mesiotemporal structures. Building on this concept, Leon‐Rojas and colleagues^35^ implemented a pipeline to automatically segment the piriform cortex and calculate chances of seizure freedom during planning. They were able to plan a new patient intervention with an estimated 50% chance of long‐term seizure freedom. More reliable and automated quantification of resected cavities may enable similar downstream applications in the future. Because ResectVol DL can list areas removed along with their volume, it can potentially help serve that purpose.

On top of the limitations already presented, we also add that our tool has not been tested on postoperative MRIs of other surgical types, such as laser interstitial thermal therapy. However, we anticipate that, due to the different profiles of lesioned brain sites, a new network should be trained on this specific dataset to accommodate these discordant characteristics. Additionally, we have not validated the labeling of resected brain structures, an area that warrants attention in future studies, and may necessitate comparisons with other segmentation methods and manual structure identification to assess the accuracy and applicability of this feature. Overall, our findings showed that ResectVol DL is an accurate and reliable tool for routine quantification of surgical cavities and for refining postoperative outcome models in epilepsy and other resective neurosurgical contexts, having failed for only one case out of 90 on which it was tested. It can be utilized in temporal and extratemporal cases and is freely available at github.com/rfcasseb/resectvol_dl/.

## 5 CONCLUSIONS

ResectVol DL successfully segmented brain cavities in MRIs of patients with epilepsy who have undergone surgery. ResectVol DL is fast, accurate, and does not require any image preprocessing, facilitating large‐cohort studies. It relies solely on postoperative 3D T1‐weighted MR images to measure cavity volumes and provides estimates of the volumes of anatomical structures within the cavity. This volumetric information may support future investigations and could potentially contribute to the development of predictive models of surgical outcomes as a downstream application.

## AUTHOR CONTRIBUTIONS

**Raphael Fernandes Casseb:** Conceptualization; methodology; software; data curation; formal analysis; visualization; writing—original draft; writing—review & editing. **Brunno Machado de Campos:** Conceptualization; methodology; software; formal analysis; writing—original draft; writing—review & editing; **Wallace Souza Loos:** Methodology; software; writing—review & editing. **Marcelo Eduardo Ramos Barbosa:** Data curation; formal analysis; writing—review & editing. **Marina Koutsodontis Machado Alvim**: Data curation; investigation; writing—review & editing; **Gabriel Chagas Lutfala Paulino:** Data curation; formal analysis; writing—review & editing. **Enrico Ghizoni**: Conceptualization; supervision; writing—review & editing. **Francesco Pucci**: Data curation; formal analysis; writing—review & editing. **Samuel Worrell**: Data curation; formal analysis; writing—review & editing. **Clarissa Lin Yassuda:** Data curation; formal analysis; writing—review & editing. **Roberto Medeiros de Souza:** Methodology; software; writing—review & editing. **Lara Jehi:** Conceptualization; resources; project administration; supervision; writing—review & editing. **Fernando Cendes:** Conceptualization; supervision; resources; project administration; writing—original draft; writing—review & editing.

## CONFLICT OF INTEREST STATEMENT

None of the authors has any conflict of interest to disclose. We confirm that we have read the Journal's position on issues involved in ethical publication and affirm that this report is consistent with those guidelines.

## Supporting information


**Figure S1.** Region labeling in the temporal cavity. Postoperative image (A), postoperative image with cavity mask (B), and postoperative image with labeled regions (C). Region names and associated volumetry information are shown in Table S1.
**Table S1.** Volumetric data of a temporal cavity segmented and characterized with ResectVol DL. Regions are presented in order of decreasing resection percentage.
**Table S2.** Acquisition protocol parameters for T1‐weighted magnetic resonance images. Mag, intensity of the B_0_ magnetic field; Manufac, magnetic resonance device manufacturer; Slc Think, slice thickness; TE, echo time; TR, repetition time.
**Figure S2.** Scatter plots for (A–D) all data (temporal, green dots; extratemporal, blue dots) and for (E–H) temporal data only between each segmentation method and manual delineation. Plots include the linear fit, the equation that best adjusts to the data with the standard error, and the identity reference line (y = x; dashed line).
**Table S3.** Pairwise comparison of Dice similarity coefficient (DSC) between methods. DSC differences (ΔDSC) are displayed along with confidence intervals, obtained via bootstrapping, and Bonferroni‐corrected *p*‐values (*p*_corrected_).
**Table S4.** Pairwise comparison of relative volumetric difference (RelDiff) between methods. Analogously, RelDiff differences (ΔRelDiff) are displayed along with confidence intervals, obtained via bootstrapping, and Bonferroni‐corrected *p*‐values (*p*_corrected_).
**Table S5.** Pairwise comparison of Pearson correlation coefficient (*r*) between methods. Correlation differences (Δ*r*) are displayed along with confidence intervals obtained via Fisher *z* transformation and Bonferroni‐corrected *p*‐values (*p*_corrected_).

## Data Availability

The data supporting the findings of this study can be made available upon approval from the ethics committee and through a formal data‐sharing agreement. ResectVol DL is available at github.com/rfcasseb/resectvol_dl/. Two anonymized T1‐weighted MR images from the test set are available for readers to test the tool at https://drive.google.com/drive/u/2/folders/18y7ObOy5DYEpQ3fZw5tAxp7NHSUOVhT3.
