# Supplementary material for: Fully automated three‐dimensional deep learning‐based magnetic resonance imaging segmentation of brain cavities in epilepsy surgery
Source: Epilepsia. 2026 Jun 12;67(8):4207–18. doi: 10.1002/epi.70296 (PMC13525573; doi:10.1002/epi.70296)
Supplement: Supplementary file 1 [file EPI-67-4207-s001.docx]

# S1. Structure identification

After segmenting the cavity, ResectVol DL combines the cavity mask with the brain-extracted image to estimate the original 3D brain shape. This recreated whole brain undergoes a series of registration steps that register a labeled template in the Montreal Neurological Institute (MNI) space onto the space of the original image (native). We use the Desikan-Killiany-Tourville (DKT) atlas^1^ as a reference for naming brain structures. Our pipeline uses functions from the Advanced Normalization Tools (ANTs) package^2^, the Statistical Parametric Mapping (SPM) toolbox^3^, and the FMRIB Software Library (FSL)^4^. **Figure S1** illustrates an example of the labeled regions of a temporal patient along with the table containing volumetry information (**Table S1**).

***Figure S1.*** *Region labeling in the temporal cavity. Postoperative image (****A****); postoperative image with cavity mask (****B****); and postoperative image with labeled regions(****C****). Region names and associated volumetry information are shown in* ***Table S1****.*

| ***Table S1.*** *Volumetric data of a temporal cavity segmented and characterized with ResectVol DL. Regions are presented in order of decreasing resection percentage.* | | | | |
| --- | --- | --- | --- | --- |
| **Cluster 1:** |  |  |  |  |
| **Volume: 253194 voxels (42594.15 mm3)** | |  |  |  |
| **ROI Index** | **ROI Name** | **Resected vol (voxel)** | **Resected vol (mm3)** | **Resected %** |
| 1006 | Left Entorhinal Cortex (Gray Matter) | 6504 | 1094.15 | 81 |
| 17 | Left Hippocampus | 14644 | 2463.52 | 69 |
| 18 | Left Amygdala | 4304 | 724.05 | 67 |
| 1016 | Left Parahippocampal Gyrus (Gray Matter) | 3535 | 594.68 | 50 |
| 1009 | Left Inferior Temporal Gyrus (Gray Matter) | 29956 | 5039.42 | 47 |
| 1015 | Left Middle Temporal Gyrus (Gray Matter) | 28314 | 4763.19 | 36 |
| 1030 | Left Superior Temporal Gyrus (Gray Matter) | 29696 | 4995.68 | 31 |
| 1007 | Left Fusiform Gyrus (Gray Matter) | 8938 | 1503.62 | 24 |
| 2 | White Matter of the Left Cerebral Hemisphere | 101315 | 17043.95 | 7 |
| 1012 | Left Lateral Orbital Gyrus (Gray Matter) | 638 | 107.33 | 1 |
| 1035 | Left Insula (Gray Matter) | 92 | 15.48 | 0 |
| 77 | White Matter Hypointensities | 15 | 2.52 | 0 |
| 1013 | Left Lingual Gyrus (Gray Matter) | 11 | 1.85 | 0 |
| 999 | Undetermined | 25232 | 4244.71 | --- |

# S2. Acquisition protocols

| ***Table S2.*** *Acquisition protocol parameters for T1-weighted MR images. Slc Think: slice thickness; Mag: intensity of the B_0_ magnetic field; TR: repetition time; TE: echo time; Manufac: MR device manufacturer.* | | | | | | | | | | | | | |
| --- | --- | --- | --- | --- | --- | --- | --- | --- | --- | --- | --- | --- | --- |
| **Subj** | **Set** | **Matrix** | | | **Voxel Size (mm)** | | **Slc Thick (mm)** | **Slices** | **Mag (T)** | **TR (ms)** | **TE (ms)** | **Manufac.** | **Model** |
| 1 | Train | 256 | 170 | 1.00 | | 1.00 | 1.00 | 256 | 3 | 9 | 2.3 | Philips | Achieva |
| 2 | Train | 180 | 240 | 1.00 | | 1.00 | 1.00 | 240 | 3 | 7 | 3.2 | Philips | Achieva |
| 3 | Train | 180 | 240 | 1.00 | | 1.00 | 1.00 | 240 | 3 | 7 | 3.2 | Philips | Achieva |
| 4 | Train | 180 | 240 | 1.00 | | 1.00 | 1.00 | 240 | 3 | 7 | 3.2 | Philips | Achieva |
| 5 | Train | 180 | 240 | 1.00 | | 1.00 | 1.00 | 240 | 3 | 7 | 3.2 | Philips | Achieva |
| 6 | Train | 256 | 180 | 1.00 | | 1.00 | 1.00 | 256 | 3 | 8 | 3.7 | Philips | Achieva |
| 7 | Train | 180 | 240 | 1.00 | | 1.00 | 1.00 | 240 | 3 | 7 | 3.2 | Philips | Achieva |
| 8 | Train | 180 | 240 | 1.00 | | 1.00 | 1.00 | 240 | 3 | 8 | 3.7 | Philips | Achieva |
| 9 | Train | 180 | 240 | 1.00 | | 1.00 | 1.00 | 240 | 3 | 7 | 3.2 | Philips | Achieva |
| 10 | Train | 180 | 240 | 1.00 | | 1.00 | 1.00 | 240 | 3 | 7 | 3.2 | Philips | Achieva |
| 11 | Train | 356 | 512 | 0.45 | | 0.45 | 0.45 | 512 | 3 | 7 | 3.2 | Philips | Achieva |
| 12 | Train | 180 | 240 | 1.00 | | 1.00 | 1.00 | 240 | 3 | 7 | 3.2 | Philips | Achieva |
| 13 | Train | 180 | 240 | 1.00 | | 1.00 | 1.00 | 240 | 3 | 7 | 3.2 | Philips | Achieva |
| 14 | Train | 180 | 240 | 1.00 | | 1.00 | 1.00 | 240 | 3 | 7 | 3.2 | Philips | Achieva |
| 15 | Train | 180 | 240 | 1.00 | | 1.00 | 1.00 | 240 | 3 | 7 | 3.2 | Philips | Achieva |
| 16 | Train | 180 | 240 | 1.00 | | 1.00 | 1.00 | 240 | 3 | 7 | 3.2 | Philips | Achieva |
| 17 | Train | 180 | 240 | 1.00 | | 1.00 | 1.00 | 240 | 3 | 7 | 3.2 | Philips | Achieva |
| 18 | Train | 180 | 240 | 1.00 | | 1.00 | 1.00 | 240 | 3 | 7 | 3.2 | Philips | Achieva |
| 19 | Train | 180 | 240 | 1.00 | | 1.00 | 1.00 | 240 | 3 | 7 | 3.2 | Philips | Achieva |
| 20 | Train | 180 | 240 | 1.00 | | 1.00 | 1.00 | 240 | 3 | 7 | 3.2 | Philips | Achieva |
| 21 | Train | 180 | 240 | 1.00 | | 1.00 | 1.00 | 240 | 3 | 7 | 3.2 | Philips | Achieva |
| 22 | Train | 180 | 240 | 1.00 | | 1.00 | 1.00 | 240 | 3 | 7 | 3.2 | Philips | Achieva |
| 23 | Train | 180 | 240 | 1.00 | | 1.00 | 1.00 | 240 | 3 | 7 | 3.2 | Philips | Achieva |
| 24 | Train | 256 | 160 | 0.90 | | 1.25 | 0.90 | 256 | 1.5 | 11 | 4.9 | Siemens | Aera |
| 25 | Train | 224 | 192 | 0.90 | | 1.25 | 0.90 | 256 | 1.5 | 11 | 4.9 | Siemens | Aera |
| 26 | Train | 224 | 192 | 0.90 | | 1.25 | 0.90 | 256 | 1.5 | 11 | 4.9 | Siemens | Aera |
| 27 | Train | 256 | 160 | 0.90 | | 1.25 | 0.90 | 256 | 1.5 | 11 | 4.6 | Siemens | Avanto |
| 28 | Train | 224 | 192 | 0.90 | | 1.25 | 0.90 | 256 | 1.5 | 11 | 4.6 | Siemens | Avanto |
| 29 | Train | 256 | 160 | 0.90 | | 1.25 | 0.90 | 256 | 1.5 | 11 | 4.6 | Siemens | Avanto |
| 30 | Train | 256 | 256 | 1.00 | | 1.00 | 1.00 | 160 | 1.5 | 11 | 4.6 | Siemens | Avanto |
| 31 | Train | 224 | 160 | 0.90 | | 1.50 | 0.90 | 256 | 1.5 | 11 | 4.6 | Siemens | Avanto |
| 32 | Train | 256 | 192 | 0.90 | | 1.25 | 0.90 | 256 | 1.5 | 11 | 4.6 | Siemens | Avanto |
| 33 | Train | 208 | 192 | 0.90 | | 1.25 | 0.90 | 256 | 1.5 | 11 | 4.6 | Siemens | Avanto |
| 34 | Train | 224 | 160 | 0.90 | | 1.50 | 0.90 | 256 | 1.5 | 11 | 4.6 | Siemens | Avanto |
| 35 | Train | 256 | 160 | 0.90 | | 1.25 | 0.90 | 256 | 1.5 | 11 | 4.6 | Siemens | Avanto |
| 36 | Train | 216 | 192 | 1.00 | | 1.25 | 1.00 | 256 | 1.5 | 11 | 4.6 | Siemens | Avanto |
| 37 | Train | 256 | 192 | 0.90 | | 1.25 | 0.90 | 256 | 1.5 | 11 | 4.6 | Siemens | Avanto |
| 38 | Train | 256 | 256 | 1.00 | | 1.00 | 1.00 | 192 | 1.5 | 11 | 4.6 | Siemens | Avanto |
| 39 | Train | 256 | 176 | 0.90 | | 1.50 | 0.90 | 256 | 1.5 | 11 | 4.6 | Siemens | Avanto |
| 40 | Train | 256 | 192 | 0.90 | | 1.25 | 0.90 | 256 | 1.5 | 11 | 4.6 | Siemens | Avanto |
| 41 | Train | 256 | 256 | 0.82 | | 1.00 | 0.82 | 256 | 3 | 1800 | 2.7 | Siemens | Biograph_mMR |
| 42 | Train | 256 | 256 | 0.82 | | 1.00 | 0.82 | 256 | 3 | 1800 | 2.7 | Siemens | Biograph_mMR |
| 43 | Train | 256 | 192 | 0.90 | | 1.25 | 0.90 | 256 | 1.5 | 11 | 4.6 | Siemens | Espree |
| 44 | Train | 256 | 192 | 0.90 | | 1.25 | 0.90 | 256 | 1.5 | 11 | 4.6 | Siemens | Espree |
| 45 | Train | 256 | 192 | 0.90 | | 1.25 | 0.90 | 256 | 1.5 | 11 | 4.6 | Siemens | Espree |
| 46 | Train | 256 | 192 | 0.90 | | 1.25 | 0.90 | 256 | 1.5 | 11 | 4.6 | Siemens | Espree |
| 47 | Train | 224 | 160 | 0.90 | | 1.25 | 0.90 | 256 | 1.5 | 14 | 3.8 | Siemens | Espree |
| 48 | Train | 256 | 192 | 0.90 | | 1.25 | 0.90 | 256 | 1.5 | 11 | 4.6 | Siemens | Espree |
| 49 | Train | 256 | 192 | 0.90 | | 1.25 | 0.90 | 256 | 1.5 | 11 | 4.6 | Siemens | Espree |
| 50 | Train | 256 | 128 | 1.00 | | 2.00 | 1.00 | 256 | 1.5 | 1990 | 3.2 | Siemens | Espree |
| 51 | Train | 512 | 256 | 0.41 | | 1.00 | 0.41 | 512 | 3 | 1800 | 2.6 | Siemens | Skyra |
| 52 | Train | 512 | 256 | 0.41 | | 1.00 | 0.41 | 512 | 3 | 1800 | 2.6 | Siemens | Skyra |
| 53 | Train | 512 | 256 | 0.41 | | 1.00 | 0.41 | 512 | 3 | 1800 | 2.6 | Siemens | Skyra |
| 54 | Train | 512 | 256 | 0.41 | | 1.00 | 0.41 | 512 | 3 | 1800 | 2.6 | Siemens | Skyra |
| 55 | Train | 512 | 256 | 0.41 | | 1.00 | 0.41 | 512 | 3 | 1800 | 2.6 | Siemens | Skyra |
| 56 | Train | 512 | 256 | 0.41 | | 1.00 | 0.41 | 512 | 3 | 1800 | 2.6 | Siemens | Skyra |
| 57 | Train | 512 | 256 | 0.41 | | 1.00 | 0.41 | 512 | 3 | 1800 | 2.6 | Siemens | Skyra |
| 58 | Train | 512 | 256 | 0.41 | | 1.00 | 0.41 | 512 | 3 | 1800 | 2.6 | Siemens | Skyra |
| 59 | Train | 512 | 256 | 0.41 | | 1.00 | 0.41 | 512 | 3 | 1800 | 2.6 | Siemens | Skyra |
| 60 | Train | 512 | 256 | 0.41 | | 1.00 | 0.41 | 512 | 3 | 1800 | 2.6 | Siemens | Skyra |
| 61 | Train | 512 | 256 | 0.41 | | 1.00 | 0.41 | 512 | 3 | 1800 | 2.6 | Siemens | Skyra |
| 62 | Train | 512 | 256 | 0.41 | | 1.00 | 0.41 | 512 | 3 | 1800 | 2.6 | Siemens | Skyra |
| 63 | Train | 512 | 256 | 0.41 | | 1.00 | 0.41 | 512 | 3 | 1800 | 2.6 | Siemens | Skyra |
| 64 | Train | 256 | 192 | 1.00 | | 1.50 | 1.00 | 256 | 3 | 11 | 4.7 | Siemens | Skyra |
| 65 | Train | 512 | 240 | 0.41 | | 1.00 | 0.41 | 512 | 3 | 1800 | 2.6 | Siemens | Skyra |
| 66 | Train | 384 | 96 | 0.41 | | 2.00 | 0.41 | 512 | 1.5 | 1850 | 3.9 | Siemens | Symphony |
| 67 | Train | 256 | 176 | 0.82 | | 1.25 | 0.82 | 256 | 1.5 | 1820 | 4.4 | Siemens | Symphony |
| 68 | Train | 256 | 176 | 0.78 | | 1.25 | 0.78 | 256 | 1.5 | 1920 | 4.4 | Siemens | Symphony |
| 69 | Train | 256 | 128 | 1.00 | | 2.00 | 1.00 | 256 | 1.5 | 1990 | 3.9 | Siemens | Symphony |
| 70 | Train | 256 | 288 | 0.82 | | 1.00 | 0.82 | 256 | 3 | 1800 | 2.4 | Siemens | TrioTim |
| 71 | Train | 256 | 288 | 0.82 | | 1.00 | 0.82 | 256 | 3 | 1800 | 2.4 | Siemens | TrioTim |
| 72 | Train | 256 | 144 | 0.82 | | 1.25 | 0.82 | 256 | 3 | 2100 | 2.9 | Siemens | TrioTim |
| 73 | Test | 180 | 240 | 1.00 | | 1.00 | 1.00 | 240 | 3 | 7 | 3.2 | Philips | Achieva |
| 74 | Test | 180 | 240 | 1.00 | | 1.00 | 1.00 | 240 | 3 | 7 | 3.2 | Philips | Achieva |
| 75 | Test | 180 | 240 | 1.00 | | 1.00 | 1.00 | 240 | 3 | 7 | 3.2 | Philips | Achieva |
| 76 | Test | 180 | 240 | 1.00 | | 1.00 | 1.00 | 240 | 3 | 7 | 3.2 | Philips | Achieva |
| 77 | Test | 180 | 240 | 1.00 | | 1.00 | 1.00 | 240 | 3 | 7 | 3.2 | Philips | Achieva |
| 78 | Test | 180 | 240 | 1.00 | | 1.00 | 1.00 | 240 | 3 | 7 | 3.2 | Philips | Achieva |
| 79 | Test | 180 | 240 | 1.00 | | 1.00 | 1.00 | 240 | 3 | 7 | 3.2 | Philips | Achieva |
| 80 | Test | 180 | 240 | 1.00 | | 1.00 | 1.00 | 240 | 3 | 7 | 3.2 | Philips | Achieva |
| 81 | Test | 180 | 240 | 1.00 | | 1.00 | 1.00 | 240 | 3 | 7 | 3.2 | Philips | Achieva |
| 82 | Test | 224 | 192 | 0.90 | | 1.25 | 0.90 | 256 | 1.5 | 11 | 4.9 | Siemens | Aera |
| 83 | Test | 224 | 192 | 0.90 | | 1.25 | 0.90 | 256 | 1.5 | 11 | 4.9 | Siemens | Aera |
| 84 | Test | 224 | 192 | 0.90 | | 1.25 | 0.90 | 256 | 1.5 | 11 | 4.9 | Siemens | Aera |
| 85 | Test | 224 | 192 | 0.90 | | 1.25 | 0.90 | 256 | 1.5 | 11 | 4.9 | Siemens | Aera |
| 86 | Test | 224 | 192 | 0.90 | | 1.25 | 0.90 | 256 | 1.5 | 11 | 4.6 | Siemens | Avanto |
| 87 | Test | 256 | 192 | 0.90 | | 1.25 | 0.90 | 256 | 1.5 | 11 | 4.6 | Siemens | Avanto |
| 88 | Test | 224 | 160 | 0.90 | | 1.50 | 0.90 | 256 | 1.5 | 11 | 4.6 | Siemens | Avanto |
| 89 | Test | 256 | 176 | 0.90 | | 1.25 | 0.90 | 256 | 1.5 | 11 | 4.6 | Siemens | Avanto |
| 90 | Test | 256 | 192 | 0.90 | | 1.25 | 0.90 | 256 | 1.5 | 11 | 4.6 | Siemens | Avanto |
| 91 | Test | 216 | 192 | 1.00 | | 1.25 | 1.00 | 256 | 1.5 | 11 | 4.6 | Siemens | Avanto |
| 92 | Test | 224 | 160 | 0.90 | | 1.50 | 0.90 | 256 | 1.5 | 11 | 4.6 | Siemens | Avanto |
| 93 | Test | 256 | 160 | 0.90 | | 1.25 | 0.90 | 256 | 1.5 | 11 | 4.6 | Siemens | Avanto |
| 94 | Test | 256 | 256 | 0.82 | | 1.00 | 0.82 | 256 | 3 | 1800 | 2.7 | Siemens | Biograph_mMR |
| 95 | Test | 256 | 192 | 0.98 | | 1.50 | 0.98 | 256 | 3 | 11 | 5.0 | Siemens | Biograph_mMR |
| 96 | Test | 256 | 256 | 0.82 | | 1.00 | 0.82 | 256 | 3 | 1800 | 2.7 | Siemens | Biograph_mMR |
| 97 | Test | 256 | 256 | 0.82 | | 1.00 | 0.82 | 256 | 3 | 1800 | 2.7 | Siemens | Biograph_mMR |
| 98 | Test | 256 | 192 | 0.90 | | 1.25 | 0.90 | 256 | 1.5 | 11 | 4.6 | Siemens | Espree |
| 99 | Test | 256 | 192 | 0.90 | | 1.25 | 0.90 | 256 | 1.5 | 11 | 4.6 | Siemens | Espree |
| 100 | Test | 256 | 192 | 0.90 | | 1.25 | 0.90 | 256 | 1.5 | 11 | 4.6 | Siemens | Espree |
| 101 | Test | 512 | 256 | 0.41 | | 1.00 | 0.41 | 512 | 3 | 1800 | 2.6 | Siemens | Skyra |
| 102 | Test | 512 | 256 | 0.41 | | 1.00 | 0.41 | 512 | 3 | 1800 | 2.6 | Siemens | Skyra |
| 103 | Test | 512 | 256 | 0.41 | | 1.00 | 0.41 | 512 | 3 | 1800 | 2.6 | Siemens | Skyra |
| 104 | Test | 512 | 256 | 0.41 | | 1.00 | 0.41 | 512 | 3 | 1800 | 2.6 | Siemens | Skyra |
| 105 | Test | 512 | 256 | 0.41 | | 1.00 | 0.41 | 512 | 3 | 1800 | 2.6 | Siemens | Skyra |
| 106 | Test | 512 | 256 | 0.41 | | 1.00 | 0.41 | 512 | 3 | 1800 | 2.6 | Siemens | Skyra |
| 107 | Test | 512 | 256 | 0.41 | | 1.00 | 0.41 | 512 | 3 | 1800 | 2.6 | Siemens | Skyra |
| 108 | Test | 512 | 256 | 0.41 | | 1.00 | 0.41 | 512 | 3 | 1800 | 2.6 | Siemens | Skyra |
| 109 | Test | 512 | 256 | 0.41 | | 1.00 | 0.41 | 512 | 3 | 1800 | 2.6 | Siemens | Skyra |
| 110 | Test | 512 | 256 | 0.41 | | 1.00 | 0.41 | 512 | 3 | 1800 | 2.6 | Siemens | Skyra |
| 111 | Test | 512 | 256 | 0.41 | | 1.00 | 0.41 | 512 | 3 | 1800 | 2.6 | Siemens | Skyra |
| 112 | Test | 512 | 256 | 0.41 | | 1.00 | 0.41 | 512 | 3 | 1800 | 2.6 | Siemens | Skyra |
| 113 | Test | 512 | 256 | 0.41 | | 1.00 | 0.41 | 512 | 3 | 1800 | 2.6 | Siemens | Skyra |
| 114 | Test | 512 | 256 | 0.41 | | 1.00 | 0.41 | 512 | 3 | 1800 | 2.6 | Siemens | Skyra |
| 115 | Test | 512 | 256 | 0.41 | | 1.00 | 0.41 | 512 | 3 | 1800 | 2.6 | Siemens | Skyra |
| 116 | Test | 512 | 256 | 0.41 | | 1.00 | 0.41 | 512 | 3 | 1800 | 2.6 | Siemens | Skyra |
| 117 | Test | 512 | 256 | 0.41 | | 1.00 | 0.41 | 512 | 3 | 2200 | 2.5 | Siemens | Skyra |
| 118 | Test | 512 | 256 | 0.41 | | 1.00 | 0.41 | 512 | 3 | 1800 | 2.6 | Siemens | Skyra |
| 119 | Test | 512 | 256 | 0.41 | | 1.00 | 0.41 | 512 | 3 | 1800 | 2.6 | Siemens | Skyra |
| 120 | Test | 512 | 256 | 0.41 | | 1.00 | 0.41 | 512 | 3 | 1800 | 2.6 | Siemens | Skyra |
| 121 | False Pos. | 180 | 240 | 1.00 | | 1.00 | 1.00 | 240 | 3 | 7 | 3.2 | Philips | Achieva |
| 122 | False Pos. | 180 | 240 | 1.00 | | 1.00 | 1.00 | 240 | 3 | 7 | 3.2 | Philips | Achieva |
| 123 | False Pos. | 180 | 240 | 1.00 | | 1.00 | 1.00 | 240 | 3 | 7 | 3.2 | Philips | Achieva |
| 124 | False Pos. | 180 | 240 | 1.00 | | 1.00 | 1.00 | 240 | 3 | 7 | 3.2 | Philips | Achieva |
| 125 | False Pos. | 180 | 240 | 1.00 | | 1.00 | 1.00 | 240 | 3 | 7 | 3.2 | Philips | Achieva |
| 126 | False Pos. | 180 | 240 | 1.00 | | 1.00 | 1.00 | 240 | 3 | 7 | 3.2 | Philips | Achieva |
| 127 | False Pos. | 180 | 240 | 1.00 | | 1.00 | 1.00 | 240 | 3 | 7 | 3.2 | Philips | Achieva |
| 128 | False Pos. | 180 | 240 | 1.00 | | 1.00 | 1.00 | 240 | 3 | 7 | 3.2 | Philips | Achieva |
| 129 | False Pos. | 180 | 240 | 1.00 | | 1.00 | 1.00 | 240 | 3 | 7 | 3.2 | Philips | Achieva |
| 130 | False Pos. | 180 | 240 | 1.00 | | 1.00 | 1.00 | 240 | 3 | 7 | 3.2 | Philips | Achieva |
| 131 | False Pos. | 180 | 240 | 1.00 | | 1.00 | 1.00 | 240 | 3 | 7 | 3.2 | Philips | Achieva |
| 132 | False Pos. | 180 | 240 | 1.00 | | 1.00 | 1.00 | 240 | 3 | 7 | 3.2 | Philips | Achieva |
| 133 | False Pos. | 180 | 240 | 1.00 | | 1.00 | 1.00 | 240 | 3 | 7 | 3.2 | Philips | Achieva |
| 134 | False Pos. | 180 | 240 | 1.00 | | 1.00 | 1.00 | 240 | 3 | 7 | 3.2 | Philips | Achieva |
| 135 | False Pos. | 180 | 240 | 1.00 | | 1.00 | 1.00 | 240 | 3 | 7 | 3.2 | Philips | Achieva |
| 136 | False Pos. | 180 | 240 | 1.00 | | 1.00 | 1.00 | 240 | 3 | 7 | 3.2 | Philips | Achieva |
| 137 | False Pos. | 180 | 240 | 1.00 | | 1.00 | 1.00 | 240 | 3 | 7 | 3.2 | Philips | Achieva |
| 138 | False Pos. | 180 | 240 | 1.00 | | 1.00 | 1.00 | 240 | 3 | 7 | 3.2 | Philips | Achieva |
| 139 | False Pos. | 180 | 240 | 1.00 | | 1.00 | 1.00 | 240 | 3 | 7 | 3.2 | Philips | Achieva |
| 140 | False Pos. | 180 | 240 | 1.00 | | 1.00 | 1.00 | 240 | 3 | 7 | 3.2 | Philips | Achieva |
| 141 | False Pos. | 180 | 240 | 1.00 | | 1.00 | 1.00 | 240 | 3 | 7 | 3.2 | Philips | Achieva |
| 142 | False Pos. | 180 | 240 | 1.00 | | 1.00 | 1.00 | 240 | 3 | 7 | 3.2 | Philips | Achieva |
| 143 | Brain Tumor | 180 | 240 | 1.00 | | 1.00 | 1.00 | 240 | 3 | 7 | 3.2 | Philips | Achieva |
| 144 | Brain Tumor | 180 | 240 | 1.00 | | 1.00 | 1.00 | 240 | 3 | 7 | 3.2 | Philips | Achieva |
| 145 | Brain Tumor | 180 | 240 | 1.00 | | 1.00 | 1.00 | 240 | 3 | 7 | 3.2 | Philips | Achieva |
| 146 | Brain Tumor | 180 | 240 | 1.00 | | 1.00 | 1.00 | 240 | 3 | 7 | 3.2 | Philips | Achieva |
| 147 | Brain Tumor | 180 | 240 | 1.00 | | 1.00 | 1.00 | 240 | 3 | 7 | 3.2 | Philips | Achieva |
| 148 | Brain Tumor | 180 | 240 | 1.00 | | 1.00 | 1.00 | 240 | 3 | 7 | 3.2 | Philips | Achieva |
| 149 | Brain Tumor | 180 | 240 | 1.00 | | 1.00 | 1.00 | 240 | 3 | 7 | 3.2 | Philips | Achieva |
| 150 | Brain Tumor | 180 | 240 | 1.00 | | 1.00 | 1.00 | 240 | 3 | 7 | 3.2 | Philips | Achieva |
| 151 | Brain Tumor | 180 | 240 | 1.00 | | 1.00 | 1.00 | 240 | 3 | 7 | 3.2 | Philips | Achieva |
| 152 | Brain Tumor | 180 | 240 | 1.00 | | 1.00 | 1.00 | 240 | 3 | 7 | 3.2 | Philips | Achieva |
| 153 | Brain Tumor | 180 | 240 | 1.00 | | 1.00 | 1.00 | 240 | 3 | 7 | 3.2 | Philips | Achieva |
| 154 | Brain Tumor | 180 | 240 | 1.00 | | 1.00 | 1.00 | 240 | 3 | 7 | 3.2 | Philips | Achieva |
| 155 | Brain Tumor | 180 | 240 | 1.00 | | 1.00 | 1.00 | 240 | 3 | 7 | 3.2 | Philips | Achieva |
| 156 | Brain Tumor | 180 | 240 | 1.00 | | 1.00 | 1.00 | 240 | 3 | 7 | 3.2 | Philips | Achieva |
| 157 | Brain Tumor | 180 | 240 | 1.00 | | 1.00 | 1.00 | 240 | 3 | 7 | 3.2 | Philips | Achieva |
| 158 | Brain Tumor | 180 | 240 | 1.00 | | 1.00 | 1.00 | 240 | 3 | 7 | 3.2 | Philips | Achieva |
| 159 | Brain Tumor | 180 | 240 | 1.00 | | 1.00 | 1.00 | 240 | 3 | 7 | 3.2 | Philips | Achieva |
| 160 | Brain Tumor | 180 | 240 | 1.00 | | 1.00 | 1.00 | 240 | 3 | 7 | 3.2 | Philips | Achieva |
| 161 | Brain Tumor | 180 | 240 | 1.00 | | 1.00 | 1.00 | 240 | 3 | 7 | 3.2 | Philips | Achieva |
| 162 | Brain Tumor | 180 | 240 | 1.00 | | 1.00 | 1.00 | 240 | 3 | 7 | 3.2 | Philips | Achieva |

# S3. Assessment of manual cavity segmentation on postoperative images

Manual segmentation of resection cavities is generally regarded as the obvious first approach and arguably yields the best quality results. However, it is susceptible to human error and consistency factors, such as inter- and intra-rater variability. In this study, five raters manually segmented subsets of the postoperative MR images. A subset of 48 extratemporal images was segmented more than once^[1]^, enabling the assessment of inter-rater agreement. We found a median inter-rater DSC of .88 (Q1-Q3: .78 - .91; range: .00 - .97), which is comparable to previous reports on brain tumors (.84 - .86) ^5^ and cavity resection (.84) ^6^.

Regarding the duration of the manual segmentation task, three raters timed the delineation, yielding approximate median times of 114 minutes (MERB, n = 31); 70 minutes (RFC, n = 14); and 54 minutes (GCLP, n = 19). Raters spent more time in our study (approximately or more than 1 hour) than what has been reported by other authors (approximately 30 minutes) ^7,8^. We also found that duration depends on lesion complexity (as reported in these studies), the tool used, and the rater's experience.

# S4. Performance of other approaches

The test set of 48 images with resective cavities and the additional set of 22 no-surgery healthy controls were also processed by ResectVol 1.1.2, DeepResection, and Auto3DSeg.

ResectVol 1.1.2 requires no pre-processing of the images, although the origin of image space (x=0, y=0, z=0) must not be too far off the anterior commissure, in which case reorienting is necessary. It took, on average, 9 minutes and 17 seconds to process each dataset, which consisted of pre- and postoperative images. We found a median DSC = .775 (Q1-Q3: .651 - .835), a correlation between the manual and automatic volumes of r(46) = .628 (p < .001), and a median relative difference of 44.0% (Q1-Q3: 29.4 – 74.6%). For the 22 control images, it incorrectly identified a cavity in all cases (volume range: .3 - 8.9 cm³) (**Table 2** and **Figure S2**).

Like the other tools, DeepResection does not require any image preprocessing. The authors do recommend, however, that images are in Left-Anterior-Superior orientation because predictions can be inaccurate otherwise. We used *fslorient* function from the FMRIB Software Library (FSL) ^4^ to perform the reorientation. The available version of DeepResection is only intended for temporal resections; thus, the results for this tool are mostly restricted to this brain region. Temporal cavity predictions overlapped with the manual segmentation in all cases, yielding a DSC = .898 (Q1-Q3: .858 – .919). The correlation and relative difference were r(46) = .940 (p < .001) and 12.5% (Q1-Q3: 6.3 – 19.6%), respectively (**Table 2** and **Figure S2**).

Auto3DSeg is a self-configuring framework that requires minimal user intervention and no prior image preprocessing. It automatically applies data augmentation (see ^9^ for details) and performs hyper-parameter tuning for the three included architectures—DiNTS, SegResNet, and Swin UNETR—using five-fold cross-validation for each network (15 trained models in total). The five best models are then combined into an ensemble for inference. In our study, the final ensemble we obtained after training on our dataset comprised one DiNTS and four SegResNet models. Inference on each of the 48 test images took approximately 39 seconds and achieved a median DSC of .919 (.883 – .945), failing only for the smallest cavity in the test sets (volume = 494.6 mm^3^), like ResectVol DL. The correlation between automatic and manual volume was r(46) = .988 (p < .001), with a median relative volume difference of 8.4% (Q1-Q3: 3.3 – 16.8%). Auto3DSeg produced false-positive cavities in 3 of the 22 no-surgery control cases (volumes: 1, 5, and 505 mm^3^).

***Figure S2.*** *Scatter plots for (****A-D****) all data (temporal – green dots; extratemporal – blue dots) and for (****E-H****) temporal data only between each segmentation method and manual delineation. Plots include the linear fit, the equation that best adjusts to the data with the standard error, and the identity reference line (y = x; dashed line).*

Significance tests on DSC, relative volumetric difference and correlation are exhibited in **Tables S3**, **S4**, and **S5**. Overall, DSCs were different across all methods, except for Auto3DSeg and ResectVol DL in the extratemporal group (**Table S3**). Regarding relative volumetric differences, ResectVol DL and Auto3DSeg exhibited no difference in any of the pairwise comparisons, while all other pairs were significantly different (**Table S4**). Finally, tests for correlation values revealed that only ResectVol 1.1.2 showed significant differences to all other methods, except for the extratemporal data, when compared against ResectVol DL (**Table S5**).

| ***Table S3.*** *Pairwise comparison of Dice similarity coefficient (DSC) between methods. DSC differences (ΔDSC) are displayed along with confidence intervals, obtained via bootstrapping, and Bonferroni-corrected p-values (p_corrected_).* | | | | | | | |
| --- | --- | --- | --- | --- | --- | --- | --- |
|  | | ***All epilepsy data*** | | ***Temporal data only*** | | ***Extratemporal data only*** | |
| **Method A** | **Method B** | **ΔDSC (B-A)** | **p_corrected_** | **ΔDSC (B-A)** | **p_corrected_** | **ΔDSC (B-A)** | **p_corrected_** |
| *Auto3DSeg* | *ResectVol DL* | .004 [.002 - .007] | .024 | .005 [.004 - .008] | .022 | .003 [-.004 - .008] | 1 |
| *ResectVol 1.1.2* | *ResectVol DL* | .128 [.103 - .141] | < .001 | .122 [.103 - .138] | < .001 | .135 [.094 - .225] | < .001 |
| *ResectVol 1.1.2* | *Auto3DSeg* | .118 [.100 - .145] | < .001 | .108 [.091 - .132] | .003 | .145 [.091 - .237] | .006 |
| *DeepResection* | *ResectVol DL* | -- | -- | .029 [.025 - .039] | < .001 | -- | -- |
| *DeepResection* | *Auto3DSeg* | -- | -- | .024 [.015 - .039] | .001 | -- | -- |
| *ResectVol 1.1.2* | *DeepResection* | -- | -- | .070 [.051 - .105] | .003 | -- | -- |

| ***Table S4.*** *Pairwise comparison of relative volumetric difference (RelDiff) between methods. Analogously, RelDiff differences (ΔRelDiff) are displayed along with confidence intervals, obtained via bootstrapping, and Bonferroni-corrected p-values (p_corrected_).* | | | | | | | |
| --- | --- | --- | --- | --- | --- | --- | --- |
|  | | ***All epilepsy data*** | | ***Temporal data only*** | | ***Extratemporal data only*** | |
| **Method A** | **Method B** | **ΔRelDiff (B-A) %** | **p_corrected_** | **ΔRelDiff (B-A) %** | **p_corrected_** | **ΔRelDiff (B-A) %** | **p_corrected_** |
| *Auto3DSeg* | *ResectVol DL* | .0 [-.9 - .9] | 1 | -.7 [-1.7 - .2] | 1 | .4 [-.8 – 2.3] | 1 |
| *ResectVol 1.1.2* | *ResectVol DL* | -32.1 [-51.4 - -26.3] | < .001 | -40.4 [-56.9 - -28.8] | < .001 | -26.4 [-54.0 - -12.4] | .001 |
| *ResectVol 1.1.2* | *Auto3DSeg* | -34.8 [-51.4 - -26.0] | < .001 | -39.0 [-56.5 - -28.7] | < .001 | -32.6 [-63.2 - -14.7] | .008 |
| *DeepResection* | *ResectVol DL* | -- | -- | -5.6 [-8.4 - -3.5] | .040 | -- | -- |
| *DeepResection* | *Auto3DSeg* | -- | -- | -4.8 [-6.3 - -3.4] | .040 | -- | -- |
| *ResectVol 1.1.2* | *DeepResection* | -- | -- | -33.9 [-46.7 - -22.8] | < .001 | -- | -- |

| ***Table S5.*** *Pairwise comparison of Pearson’s correlation coefficient (r) between methods.* *Correlation differences (Δr) are displayed along with confidence intervals obtained via Fisher’s z transformation and Bonferroni-corrected p-values (p_corrected_).* | | | | | | | |
| --- | --- | --- | --- | --- | --- | --- | --- |
|  | | ***All epilepsy data*** | | ***Temporal data only*** | | ***Extratemporal data only*** | |
| **Method A** | **Method B** | **Δr (B-A)** | **p_corrected_** | **Δr (B-A)** | **p_corrected_** | **Δr (B-A)** | **p_corrected_** |
| *Auto3DSeg* | *ResectVol DL* | -.004 [-.019 - .004] | 1 | .011 [-.007 - .043] | 1 | -.005 [-.031 - .004] | 1 |
| *ResectVol 1.1.2* | *ResectVol DL* | .357 [.043 - .791] | .001 | . 262 [.040 - .668] | < .001 | .371 [.015 - .875] | .056 |
| *ResectVol 1.1.2* | *Auto3DSeg* | .360 [.047 - .796] | < .001 | .251 [.026 - .655] | .018 | .376 [.027 - .879] | .007 |
| *DeepResection* | *ResectVol DL* | -- | -- | .039 [.002 - .095] | .214 | -- | -- |
| *DeepResection* | *Auto3DSeg* | -- | -- | .028 [.004 - .065] | .072 | -- | -- |
| *ResectVol 1.1.2* | *DeepResection* | -- | -- | .223 [.010 - .600] | .163 | -- | -- |

# S5. References

1.	Klein, A. & Tourville, J. 101 labeled brain images and a consistent human cortical labeling protocol. *Front. Neurosci.* https://doi.org/10.3389/fnins.2012.00171 (2012) doi:10.3389/fnins.2012.00171.

2.	Avants, B. B. *et al.* A reproducible evaluation of ANTs similarity metric performance in brain image registration. *Neuroimage* **54**, (2011).

3.	Friston, K. J. *et al.* Statistical parametric maps in functional imaging: A general linear approach. *Hum. Brain Mapp.* **2**, 189–210 (1994).

4.	Jenkinson, M., Beckmann, C. F., Behrens, T. E. J., Woolrich, M. W. & Smith, S. M. FSL. *Neuroimage* **62**, 782–790 (2012).

5.	Ermiş, E. *et al.* Fully automated brain resection cavity delineation for radiation target volume definition in glioblastoma patients using deep learning. *Radiation Oncology* **15**, (2020).

6.	Pérez-García, F. *et al.* Simulation of Brain Resection for Cavity Segmentation Using Self-supervised and Semi-supervised Learning. in *Lecture Notes in Computer Science (including subseries Lecture Notes in Artificial Intelligence and Lecture Notes in Bioinformatics)* vol. 12263 LNCS (2020).

7.	Casseb, R. F. *et al.* ResectVol: A tool to automatically segment and characterize lacunas in brain images. *Epilepsia Open* **6**, 720–726 (2021).

8.	Gau, K. *et al.* Accuracy and practical aspects of semi- and fully automatic segmentation methods for resected brain areas. *Neuroradiology* **62**, 1637–1648 (2020).

9.	Project-MONAI/tutorials. https://github.com/Project-MONAI/tutorials/blob/main/auto3dseg/docs/algorithm_generation.md.

1. The best manual mask from each case was visually selected to compose the final set of 120 manual masks used as ground truth in the subsequent analyses.
